# The impact of BMI on clinical progress, response to treatment, and disease course in patients with differentiated thyroid cancer

**DOI:** 10.1371/journal.pone.0204668

**Published:** 2018-10-01

**Authors:** Danuta Gąsior-Perczak, Iwona Pałyga, Monika Szymonek, Artur Kowalik, Agnieszka Walczyk, Janusz Kopczyński, Katarzyna Lizis-Kolus, Tomasz Trybek, Estera Mikina, Dorota Szyska-Skrobot, Klaudia Gadawska-Juszczyk, Stefan Hurej, Artur Szczodry, Anna Słuszniak, Janusz Słuszniak, Ryszard Mężyk, Stanisław Góźdź, Aldona Kowalska

**Affiliations:** 1 Endocrinology Clinic, Holycross Cancer Centre, Kielce, Poland; 2 Department of Molecular Diagnostics, Holycross Cancer Centre, Kielce, Poland; 3 Department of Surgical Pathology, Holycross Cancer Centre, Kielce, Poland; 4 Laboratory of Tumor Markers, Holycross Cancer Centre, Kielce, Poland; 5 Department of Surgical Oncology, Holycross Cancer Centre, Kielce, Poland; 6 Cancer Epidemiology, Holycross Cancer Centre, Kielce, Poland; 7 Oncology Clinic, Holycross Cancer Centre, Kielce, Poland; 8 Faculty of Health Sciences, Jan Kochanowski University, Kielce, Poland; Universidade do Porto Faculdade de Medicina, PORTUGAL

## Abstract

**Introduction:**

Obesity is a serious health problem worldwide, particularly in developed countries. It is a risk factor for many diseases, including thyroid cancer. The relationship between obesity and prognostic factors of thyroid cancer is unclear.

**Aims:**

We sought to ascertain the relationship between body mass index (BMI) and clinicopathological features increasing the risk of poor clinical course, treatment response, and clinical outcome in patients with differentiated thyroid cancer (DTC).

**Subjects & methods:**

The study included 1181 patients with DTC (88% women and 12% men) treated at a single center from 2000 to 2016. BMI before surgery and aggressive clinicopathological features, according to the American Thyroid Initial Risk stratification system, were analyzed. The relationship between BMI and initial risk, treatment response, and final status of the disease was evaluated, incorporating the revised 2015 American Thyroid Association guidelines and the 8^th^ edition of the American Joint Committee on Cancer/Tumor-Node-Metastasis (AJCC/TNM) staging system. Patients were stratified according to the World Health Organization classification of BMI. Statistical analysis was performed using univariate and multivariate logistic regression analysis.

**Results:**

Median follow-up was 7.7 years (1–16 years). There were no significant associations between BMI and extrathyroidal extension (microscopic and gross), cervical lymph node metastasis, or distant metastasis in univariate and multivariate analyses. BMI did not affect initial risk, treatment response or disease outcome. Obesity was more prevalent in men (p = 0.035) and in patients ≥55 years old (p = 0.001). There was no statistically significant relationship between BMI and more advanced TNM stage in patients ≤55 years old (stage I vs. stage II) (p = 0.266) or in patients >55 years old (stage I–II vs. III–IV) (p = 0.877).

**Conclusions:**

Obesity is not associated with more aggressive clinicopathological features of thyroid cancer. Obesity is not a risk factor for progression to more advanced stages of disease, nor is it a prognostic factor for poorer treatment response and clinical outcome.

## Introduction

Differentiated thyroid cancer (DTC) is the most common endocrine cancer worldwide, and incidence of this cancer, especially of the papillary carcinoma (PTC) type, has been increasing for several decades [1–5]. To a large extent, this increase is related to better access to modern diagnostic imaging and biopsies, which contribute to improved detection of early stages of PTC that might have remained undiagnosed in the past [5–9]. However, some authors report an increase in the number of invasive, large, or small thyroid cancers [2, 10–12], which suggests a real increase in the incidence of thyroid cancer. Improvements in the quality of imaging studies alone cannot explain the increased incidence of DTC. Genetic and environmental factors, such as exposure to ionizing radiation and iodine consumption, as well as factors associated with lifestyle, are also associated with the increase in cancer incidence [13–15].

Obesity is one of the most common public health problems worldwide, and its incidence has been increasing steadily over the past two decades in both developed and developing countries [16]. In Poland in the last decade, the percentage of obese adults has increased by 7%, and is similar to the percentage of obese Caucasian adults in the United States [17]. Epidemiological data confirm that obesity is independently associated with an increased incidence of various solid tumors, including DTC [18–22], but at the same time, there are studies that show no connection between obesity and thyroid cancer [23–25]. Links between obesity and predictors of thyroid cancer are also uncertain [26, 27]. Despite the existence of studies demonstrating the impact of obesity on thyroid cancer, no clear mechanism explaining the link has been shown. It has been hypothesized that potential mediators may include insulin, IGF-1, cytokines, inflammation, TSH, adiponectins, leptin, and estrogens [15, 28, 29].

We sought to analyze the relationship between body mass index (BMI) and clinical and pathological characteristics increasing the risk of poor clinical course, primary treatment response, and outcome of the disease in DTC patients treated in one center in Poland.

## Materials and methods

### Patients and study design

A retrospective analysis was performed of the medical records of 2100 Caucasian patients with DTC who had undergone total thyroidectomy or lobectomy at a single center during the years 2000–2016. The following data were obtained: BMI at the time of surgery, prognostic clinicopathological features (sex, age at diagnosis, tumor diameter, multifocality, lymph node metastasis, and extrathyroidal extension), response to primary treatment, and clinical outcome of disease (remission, recurrence, or death). Patients who did not have complete BMI data, patients with a follow-up period of less than 12 months, and patients whose anti-thyroglobulin antibody (TgAb) levels were monitored with an anti-thyroglobulin (Tg) recovery test rather than a direct measurement of antibodies were excluded. The study ultimately included 1181 patients.

Postoperative Tumor Node Metastasis (TNM) staging of all included patients was re-classified according to the most recent 8^th^ edition of the American Joint Committee on Cancer (AJCC)/Union for International Cancer Control (UICC) TNM staging system and the ATA-modified initial risk stratification system (low, intermediate, and high risk of recurrence) [30, 31]. At the stage of diagnosis, clinicopathological features pNx and Mx were analyzed in detail. Subsequently pNx was clinically reclassified as N0b or N1, while Mx was reclassified as 0 or 1, according to the 8^th^ edition of the AJCC/UICC TNM staging system. All suspicious changes in Nx observed in postoperative ultrasound were verified by fine-needle biopsy with the evaluation of Tg from the aspirate, as described previously [32].

Summary of the course of the disease in the present study was dated December 31, 2016; in the case of patients who died prior to this date (22/1181, 1.9%), the state of follow-up was summarized according to the condition of the disease at the time of death.

The study plan was accepted by the Bioethics Committee at the Holycross Chamber of Physicians in Kielce, Poland. It was not necessary to obtain written informed consent from patients because the data was retrospectively obtained from patients’ medical history collected during routine diagnostic procedures during hospitalization. All patient records and information were anonymized and de-identified prior to analysis.

### Treatment protocol and patient monitoring

All patients included in the study were subjected to primary surgical treatment. The scope of surgery included lobectomy, total thyroidectomy (TT), or total thyroidectomy with central compartment lymphadenectomy. In our center, total thyroidectomy with central compartment lymphadenectomy was performed if the primary tumors were >10 mm, multiple or bilateral, or extrathyroidal, or when metastases to the lymph nodes (LN) of the central neck compartment were detected during pre-operative evaluation or surgery. We routinely performed central compartment node dissection on the primary tumor side. On the other hand, we performed bilateral central compartment node dissection when the tumor was bilateral or the LNs were enlarged on the opposite side, as demonstrated during pre-operative staging or surgery. However, the decision to remove lateral LNs depended on the pre- or intra-operative diagnosis of metastases to LNs, or a strong clinical suspicion of their involvement. Lobectomy (total excision of the entire thyroid lobe with isthmus) was performed in patients diagnosed with pre-operative unifocal PTC with a diameter of ≤10 mm, in clinical stage N0 (no lymph node metastases diagnosed in preoperative ultrasound), when there were no evident indications for bilateral surgery in the form of changes visible in the ultrasound in the contralateral lobe, and in patients who had previously undergone lobectomy and were diagnosed with low-risk thyroid cancer. TT without central compartment node dissection was performed in patients with nodular goiter who were diagnosed with PTC ≤10 mm (pT1a) after surgery if they had no evidence of cervical node metastasis in clinical N0 (no LN metastases in postoperative ultrasound) and no distant metastases, and after a careful histopathological examination of the postoperative material to exclude multifocal growth.

All patients with initial postoperative tumor stage higher than pT1aN0-xM0 qualified for radioactive iodine (I-131) treatment with a subsequent suppressive dose of levothyroxine (LT4). The standard procedure, 1100–3700 MBq I-131, was administered depending on the TNM status. Protocols for I-131 treatment and evaluation of the efficacy of primary treatment in patients treated with I-131 in our center have been described previously [33]. Evaluation of treatment response was carried out 9–12 months after administration of I-131. As we previously reported, patients with ineffective ablation, defined as focal I-131 uptake in the thyroid bed >0.1% without any other features of the disease, were treated with a second dose of I-131 and reevaluated after 9–12 months [33].

The efficacy of surgical treatment in patients with pT1aN0-xM0 who were not treated with I-131 was assessed based on a clinical examination, neck ultrasound, and levels of Tg and TgAb within 4–6 weeks after surgery, before levothyroxine was administered. Patients who received a TT underwent neck and whole body scans. When the results indicated that the primary surgery was not radical enough, patients were referred to secondary TT. Further tests were carried out every 6–12 months, depending on the risk degree of the clinical course, as previously described [34].

### Diagnostic tests and imaging

Measurements of TSH, Tg, and TgAb were all performed in the same laboratory. The testing methodology has been described in detail previously [34, 35]. The details of neck ultrasound and whole body scintigraphy procedures in our center have also been reported previously [33, 34].

### Assessment of treatment response

Patients treated with I-131 were assessed for response to initial therapy (surgery with I-131) using criteria proposed by Tuttle et al. [36], which were accepted by the ATA [31]. The response was classified as excellent, indeterminate, biochemically incomplete, or structurally incomplete. Procedures performed during the follow-up until the end of I-131 treatment and assessment of the response were described previously [33]. Patients not treated with I-131 were assessed for response to initial therapy (TT or lobectomy) using criteria proposed by Momesso and Tuttle [37]. The response was classified as excellent, indeterminate, biochemically incomplete, or structurally incomplete. Procedures performed during monitoring of the course of the disease from the end of surgical treatment to evaluation of response were described previously [34].

### Anthropometric measurements

All patients included in the study were measured for height and weight without shoes and outer clothing on the day of surgery. BMI was calculated as weight in kilograms divided by the square of height in meters (kg/m^2^). BMI values were stratified according to the World Health Organization (WHO) classification: underweight (BMI, <18.5 kg/m^2^), normal (BMI, 18.5–24.9 kg/m^2^), overweight (BMI, 25.0–29.9 kg/m^2^), and obese (BMI, ≥30.0 kg/m^2^). Obesity was then further stratified into Grade 1 obesity (BMI, 30–34.9 kg/m^2^), Grade 2 obesity (BMI, 35–39.9 kg/m^2^), and Grade 3 obesity (BMI ≥40 kg/m^2^). The relationship between BMI and clinical and pathological features, the response to primary treatment, and the outcome of the disease (recurrent/persistent disease, death) was analyzed.

### Final oncological assessment

Follow-up concluded with an oncological assessment on December 31, 2016. Based on the medical documentation, patients’ health was assessed by applying the latest ATA guidelines [31] and assigning them to groups: no evidence of disease (NED), recurrent/persistent disease, death from cancer, and death from other causes.

### Statistical analyses

Basic statistics (mean, standard deviation) were determined for continuous variables (age, BMI, tumor size, years of follow-up). Percentages were determined for discrete and ordinal variables. A t-test was applied for testing differences between means. A chi-square test was used to examine the interrelationship of pairs of features. Logistic regression (univariate and multivariate) analysis was used to examine the dependence of selected clinicopathological features from selected prognostic factors. An odds ratio (OR) with a 95% confidence interval was determined. Kaplan-Meier curves were used to analyze overall survival. P-values <0.05 were considered statistically significant. All statistical analysis was performed using MedCalc Statistical Software version 17.9.7 (MedCalc Software bvba, Ostend, Belgium; http://www.medcalc.org; 2017).

## Results

### Baseline characteristics

Clinical and pathological features of patients, tumor staging, ATA Initial Risk Stratification System, category of response and final outcome of the disease are summarized in Table 1. We stratified patients into six groups: underweight (BMI, <18.5 kg/m^2^), normal weight (BMI, 18.5–24.9 kg/m^2^), overweight (BMI, 25.0–29.9 kg/m^2^), Grade 1 obesity (BMI, 30–34.9 kg/m^2^), Grade 2 obesity (BMI, 35–39.9 kg/m^2^), and Grade 3 obesity (BMI, ≥40 kg/m^2^). The numbers of patients in each group are indicated in Table 1.

**Table 1:** Characteristics of DTC patients.

Feature	Total n = 1181 (100%)
Female (F)	1039 (88%)
Male (M)	142 (12%)
Mean age at diagnosis	51.3 years (SD ± 16.5)
female	50.1 years (SD ± 17.0)
male	56.0 years (SD ± 14.2)
Age group:*	
<55 years	682 (57.7%)
≥55 years	499 (42.3%)
Mean BMI	28.1 kg/m^2^ (±5.1)
Range	16.6–53.2 kg/m^2^
BMI group:	
Underweight	8 (0.7%)
Normal	339 (28.7%)
Overweight	436 (36.9%)
BMI obesity group	398
grade 1 (30–34.9)	275 (69.1%)
grade 2 (35–39.9)	98 (24.6%)
grade 3 (≥40)	25 (6.3%)
Mean tumor size	13.2 mm (SD ± 14.9)
range	0.5 – 130mm
Histology:	
Papillary	1117 (94.6%)
Follicular	44 (3.7%)
Hürthle cell	6 (0.5%)
Poorly differentiated (insular)	14 (1.2%)
Papillary cancer histologic subtype:	
Classic	920 (82.4%)
Follicular	173 (15.5%)
Other, non-aggressive	9 (0.8%)
Other, aggressive **	15 (1.3%)
Extrathyroidal extension:	
Negative	955 (80.9%)
Microscopic	191 (16.1%)
Gross	35 (3.0%)
Vascular invasion:	
No	1111 (94.1%)
Yes	70 (5.9%)
Multifocality:	254 (21.5%)
Tumor stage: *	
T1	953 (80.8%)
T2	102 (8.6%)
T3	108 (9.1%)
T4	18 (1.5%)
Tumor diameter:	
≤ 10 mm	746 (63.2%)
> 10 mm	435 (36.8%)
Including > 20mm	228
Lymph node metastasis: *	
N0a	481 (40.7%)
N0b	563 (47.7%)
N1	137 (11.6%)
Distant metastasis:	
M0	1160 (98.2%)
M1	21 (1.8%)
TNM Stage: *	
I	1071 (90.7%)
II	76 (6.4%)
III	12 (1.0%)
IV	22 (1.9%)
Radioactive iodine (^131^I) therapy:	
Yes	821 (69.5%)
No	360 (30.5%)
ATA Initial Risk Stratification System:	
Low	815 (69.0%)
Intermediate	312 (26.4%)
High	54 (4.6%)
Response to therapy:	
Excellent	991 (83.9%)
Indeterminate	108 (9.1%)
Biochemically incomplete	27 (2.3%)
Structurally incomplete	55 (4.7%)
Follow-up:	
Median and range	7.7 years (1–16)
Final follow-up (December 31, 2016):	
NED	1097 (94.7%)
Structurally persistent disease	21 (1.8%)
Biochemically persistent disease	41 (3.5%)
Death:	22 (1.9%)
Tumor- related	13 (1.1%)
Tumor-unrelated	9 (0.8%)

BMI: body mass index (kg/m^2^); SD: standard deviation; NED: no evidence of disease; N0a: one or more cytologically or histologically confirmed benign lymph node; N0b: no radiologic or clinical evidence of locoregional lymph node metastasis; N1: metastasis to regional lymph nodes; ATA: American Thyroid Association.

* determined by 8^th^ edition of AJCC/UICC TNM staging system

** oxyphilic, diffuse sclerosing, solid

The patients had diseases of differing severities and clinical characteristics, as specified in the Table 1. Histologically, the vast majority of tumors were papillary; in terms of clinical severity, the majority were stage pT1. Most patients received I-131 treatment, at a range of doses (1100–3700 MBq) depending on tumor stage, whereas patients with small tumors without metastasis (pT1aN0-xM0) did not. Most patients (83.9%) responded well to therapy, although 5.3% presented with features of biochemically or structurally persistent disease at the end of follow-up.

### Associations between BMI and clinicopathological features of DTC

The clinicopathological features of DTC were evaluated in relation to BMI groups (Table 2). We observed no statistically significant dependence of the primary tumor size, more aggressive DTC histopathologic type or histopathologic PTC subtype, multifocality, extrathyroidal extension (microscopic or gross), vascular invasion, lymph node metastases, distant metastases, intermediate or high risk of recurrence according to ATA, poorer response to primary treatment, or outcome of the disease in relation to BMI (all six groups).

**Table 2:** Clinicopathologic characteristics according to the six BMI groups.

Feature	Underweight BMI < 18.5 n = 8	Normal 18.5 ≤ BMI < 25 n = 339	Overweight 25 ≤ BMI < 30 n = 436	Grade 1 obesity 30 ≤ BMI < 35 n = 275	Grade 2 obesity 35 ≤ BMI < 40 n = 98	Grade 3 obesity BMI ≥ 40 n = 25	P-value
Age at diagnosis (mean ± SD)	27.8 ± 11.5 years	44.1 ± 14.2 years	52.4 ± 13.2 years	54.3 ± 10.7 years	54.6 ± 11.7 years	52.8 ± 8.9 years	<0.001
Age group:*							<0.001
<55	8 (100%)	251 (74.0%)	225 (51.6%)	140 (50.9%)	45 (45.5%)	13 (54.2%)	
≥55	0 (0%)	88 (26.0%)	211 (48.4%)	135 (49.1%)	53 (54.5%)	12 (45.8%)	
Gender:							0.007
Female	8 (100%)	315 (92.9%)	377 (86.5%)	233 (84.7%)	82 (83.7%)	24 (96%)	
Male	0 (0%)	24 (7.1%)	59 (13.5%)	42 (15.3%)	16 (16.3%)	1 (4%)	
Tumor size (mm) (mean SD)	13.5 ± 11.7	12.6 ± 14.9	12.5 ± 13.5	14.5 ± 16.2	14.6 ± 17.8	10.2 ± 6.6	0.383
Tumor size >10mm							0.679
Yes	4 (50.0%)	118 (34.8%)	156 (35.8%)	110 (40.0%)	36 (36.7%)	11 (44.0%)	
No	4 (50.0%)	221 (65.2%)	280 (64.2%)	165 (60.0%)	62 (63.3%)	14 (56.0%)	
Tumor size >20mm							0.694
Yes	1 (12.5%)	63 (18.6%)	84 (19.3%)	58 (21.1%)	20 (20.4%)	2 (8.0%)	
No	7 (87.5%)	276 (81.4%)	352 (80,7%)	217 (78.9%)	78 (79.6%)	23 (92.0%)	
Histology:							0.966
Papillary	8 (100%)	321 (94.7%)	413 (94.7%)	261 (94.9%)	89 (90.9%)	25 (100%)	
Follicular	0 (0%)	13 (3.8%)	17 (3.9%)	8 (2.9%)	6 (6.1%)	0 (0%)	
Hürthle cell	0 (0%)	2 (0.6%)	2 (0.5%)	1 (0.4%)	1 (1.0%)	0 (0%)	
Poorly differentiated (insular)	0 (0%)	3 (0.9%)	4 (0.9%)	5 (1.8%)	2 (2.0%)	0 (0%)	
PTC histologic subtype:							0.828
Classic	6 (75%)	254 (79.1%)	343 (83.1%)	215 (82.4%)	80 (89.9%)	22 (88%)	
Follicular	2 (25%)	59 (18.4%)	63 (15.3%)	39 (14.9%)	7 (7.9%)	3 (12%)	
Other, non-aggressive	0 (0%)	4 (1.2%)	2 (0.5%)	2 (0.8%)	1 (1.1%)	0 (0%)	
Other, aggressive**	0 (0%)	4 (1.2%)	5 (1.2%)	5 (1.9%)	1 (1.1%)	0 (0%)	
Multifocality:							0.164
Yes	1 (12.5%)	63 (18.6%)	89 (20.4%)	75 (27.3%)	21 (21.4%)	5 (20.0%)	
No	7 (87.5%)	276 (81.4%)	347 (79.6%)	200 (72.7%)	77 (78.6%)	20 (80.0%)	
Extrathyroidal extension:							0.608
Negative	7 (87.5%)	287 (84.6%)	347 (79.5%)	216 (78.5%)	76 (77.6%)	22 (88%)	
Microscopic	1 (12.5%)	43 (12.7%)	77 (17.7%)	50 (18.2%)	17 (17.3%)	3 (12%)	
Gross	0 (0%)	9 (2.7%)	12 (2.8%)	9 (3.3%)	5 (5.1%)	0 (0%)	
Vascular invasion:							0.411
No	8 (100%)	322 (95.0%)	403 (92.4%)	261 (94.9%)	92 (93.9%)	25 (100%)	
Yes	0 (0%)	17 (5.0%)	33 (7.6%)	14 (5.1%)	6 (6.1%)	0 (0%)	
Tumor stage:*							0.814
T1	7 (87.5%)	281 (82.9%)	349 (80.0%)	214 (77.8%)	79 (80.6%)	23 (92.0%)	
T2	1 (12.5%)	26 (7.7%)	42 (9.6%)	27 (9.8%)	5 (5.1%)	1 (4.0%)	
T3	0 (0%)	27 (8.0%)	39 (8.9%)	28 (10.2%)	13 (13.3%)	1 (4.0%)	
T4	0 (0%)	5 (1.5%)	6 (1.4%)	6 (2.2%)	1 (1.0%)	0 (0.0%)	
Lymph node metastasis:*							0.512
No (N0a or N0b)	7 (87.5%)	297 (87.6%)	387 (88.8%)	244 (88.7%)	84 (85.7%)	25 (100%)	
Yes (N1)	1 (12.5%)	42 (12.4%)	49 (11.2%)	31 (11.3%)	14 (14.3%)	0 (0.0%)	
Distant metastasis:							0.965
M0	8 (100%)	333 (98.2%)	429 (98.4%)	269 (97.8%)	96 (98.0%)	25 (100%)	
M1	0 (0.0%)	6 (1.8%)	7 (1.6%)	6 (2.2%)	2 (2.0%)	0 (0.0%)	
TNM stage:*							0.140
I	8 (100%)	323 (95.3%)	393 (90.1%)	240 (87.3%)	83 (84.8%)	24 (96.0%)	
II III	0 (0.0%) 0 (0.0%)	9 (2.7%) 3 (0.9%)	29 (6.7%) 5 (1.1%)	25 (9.1%) 3 (1.1%)	12 (12.2%) 1 (1.0%)	1 (4.0%) 0 (0.0%)	
IV	0 (0.0%)	4 (1.2%)	9 (2.1%)	7 (2.5%)	2 (2.0%)	0 (0.0%)	
Radioactive iodine therapy							0.667
Yes	5 (62.5%)	224 (66.1%)	306 (70.2%)	198 (72.0%)	70 (71.4%)	18 (72.0%)	
No	3 (37.5%)	115 (33.9%)	130 (29.8%)	77 (28.0%)	28 (28.6%)	7 (28.0%)	
ATA Initial Risk Stratification System							0.489
Low	6 (75.0%)	241 (71,1%)	302 (69.2%)	181 (65.8%)	63 (64.3%)	22 (88.0%)	
Intermediate	2 (25.0%)	86 (25.4%)	115 (26.4%)	77 (28.0%)	29 (29.6%)	3 (12.0%)	
High	0 (0.0%)	12 (3.5%)	19 (4.4%)	17 (6.2%)	6 (6.1%)	0 (0.0%)	
Response to therapy:							0.389
Excellent	7 (87.5%)	288 (85.0%)	363 (83.3%)	235 (85.5%)	74 (75.5%)	24 (96.0%)	
Indeterminate	0 (0.0%)	29 (8.5%)	41 (9.3%)	22 (8.0%)	15 (15.3%)	1 (4.0%)	
Biochemically incomplete	1 (12.5%)	6 (1.8%)	9 (2.1%)	8 (2.9%)	3 (3.1%)	0 (0.0%)	
Structurally incomplete	0 (0.0%)	16 (4.7%)	23 (5.3%)	10 (3.6%)	6 (6.1%)	0 (0.0%)	
Status of final follow-up:							0.440
Remission (NED)	7 (87.5%)	321 (95.5%)	400 (93.7%)	259 (95.6%)	86 (92.5%)	24 (100.0%)	
Recurrent/persistent disease	1 (12.5%)	15 (4.5%)	27 (6.3%)	12 (4.4%)	7 (7.5%)	0 (0.0%)	
Death	0	3	9	4	5	1	0.574
Cancer-related	0 (0.0%)	2 (66.7%)	6 (66.7%)	3 (75.0%)	2 (40.0%)	0 (0.0%)	
Cancer-unrelated	0 (0.0%)	1 (33.3%)	3 (33.3%)	1 (25.0%)	3 (60.0%)	1 (100.0%)	

BMI: body mass index (kg/m^2^); SD: standard deviation; NED: no evidence of disease; ATA: American Thyroid Association; PTC: papillary thyroid cancer; N0a: one or more cytologically or histologically confirmed benign lymph node; N0b: no radiologic or clinical evidence of locoregional lymph node metastasis; N1: metastasis to regional lymph nodes)

*determined by 8^*th*^ *edition of AJCC/UICC TNM staging system*

**oxyphilic, *diffuse sclerosing*, *solid*

We observed a statistically significant relationship between sex and BMI (p = 0.007), and age and BMI (<55 years vs. ≥55 years) (p <0.001) was found. In this study population, obesity was significantly (p = 0.035) more prevalent in men (59/142; 41.5%) than in women (339/1039; 32.5%) (S1 Table). According to the updated 8^th^ edition of AJCC/TNM staging system, BMI was not significantly associated with more advanced TNM stage in patients <55 years of age (stage I vs. stage II) (p = 0.266) or ≥55 years of age (stage I-II vs. III-IV) (p = 0.877) (S1 Dataset).

### Predictive factors for aggressive pathology, response to therapy, and outcome of DTC

We performed logistic regression analysis (univariate and multivariate) to determine the dependence of selected features of pathological aggressiveness of cancer [extrathyroidal extension, lymph node metastasis, distant metastases, and ATA Initial Risk Stratification System score (high and intermediate), treatment response, and disease outcome] on prognostic factors such as age, sex, tumor size, multifocality, and BMI (Table 3). In the univariate analysis, many prognostic factors apart from BMI had a significant impact on the analyzed clinical features. In multivariate analysis of BMI (all six groups), there was no statistically significant relationship to pathological aggressiveness (i.e. extrathyroidal extension, lymph node metastases, or distant metastases).

**Table 3:** Predictive factors for aggressive pathologic features, response to therapy, and outcome of DTC, as defined by multiple logistic regression analysis.

	Logistic regression model			
	Univariate		Multivariate	
	OR (95% CI)	P-value	OR (95% CI)	P-value
Extrathyroidal extension:				
Age (years)	1.02 (1.01–1.03)	0.001	1.01 (1.00–1.02)	0.024
Male gender	1.45 (0.96–2.19)	0.076	1.21 (0.76–1.90)	0.418
Tumor size (mm)	1.04 (1.03–1.04)	<0.001	1.03 (1.03–1.04)	<0.001
Multifocality	1.93 (1.39–2.67)	0.001	2.02 (1.44–2.84)	<0.001
BMI groups	1.12 (0.97–1.28)	0.121	1.03 (0.88–1.21)	0.712
Lymph node metastasis:*				
Age (years)	0.97 (0.96–0.98)	<0.001	0.96 (0.94–0.97)	<0.001
Male gender	2.72 (1.75–4.22)	<0.001	2.29 (1.39–3.77)	0.002
Tumor size (mm)	1.03 (1.02–1.04)	<0.001	1.04 (1.03–1.05)	<0.001
Multifocality	1.66 (1.12–2.47)	0.011	2.22 (1.45–3.40)	0.003
BMI groups	0.95 (0.79–1.23)	0.524	1.05 (0.86–1.28)	0.635
Distant metastases:				
Age (years)	1.02 (0.99–1.06)	0.167	1.01 (0.97–1.05)	0.604
Male gender	3.01 (1.15–7.89)	0.025	1.46 (0.42–4.99)	0.552
Tumor size (mm)	1.05 (1.03–1.06)	<0.001	1.04 (1.03–1.06)	<0.001
Multifocality	0.60 (0.18–2.06)	0.421	0.86 (0.24–3.12)	0.819
BMI groups	1.03 (0.67–1.56)	0.896	0.94 (0.56–1.59)	0.832
ATA risk of recurrence (high and intermediate)				
Age (years)	1.00 (0.99–1.01)	0.292	0.99 (0.98–1.01)	0.827
Male gender	2.07 (1.45–2.95)	0.001	1.67 (1.12–2.46)	0.023
Tumor size (mm)	1.07 (1.06–1.09)	<0.001	1.07 (1.06–1.09)	<0.001
Multifocality	1.54 (0.16–2.06)	0.003	1.73 (1.26–2.38)	0.008
BMI groups	1.04 (0.92–1.17)	0.506	0.98 (0.85–1.14)	0.843
Response to therapy (indeterminate, biochemically incomplete, structurally incomplete):				
Age (years) Male gender	1.00 (0.99–1.02) 2.23 (1.48–3.34)	0.413 0.001	1.00 (0.99–1.01) 1.74 (1.12–2.72)	0.907 0.014
Tumor size (mm)	1.03 (1.02–1.04)	<0.001	1.03 (1.02–1.04)	<0.001
Multifocality	1.16 (0.80–1.68)	0.426	1.21 (0.82–1.78)	0.323
BMI groups	1.04 (0.89–1.21)	0.573	1.01 (0.86–1.19)	0.905
Status of final follow-up (persistent disease; deaths):				
Age (years)	1.04 (1.02–1.06)	0.001	1.03 (1.01–1.05)	0.007
Male gender	2.31 (1.34–3.99)	0.003	1.30 (0.66–2.56)	0.445
Tumor size (mm)	1.05 (1.04–1.06)	<0.001	1.05 (1.04–1.06)	<0.001
Multifocality	1.24 (0.74–2.06)	0.419	1.32 (0.75–2.33)	0.340
BMI groups	1.12 (0.91–1.38)	0.297	0.92 (0.71–1.95)	0.534

ATA: American Thyroid Association; OR: odds ratio; CI: confidence interval; BMI groups: group 1 (underweight; BMI, <18.5 kg/m^2^), group 2 (normal; BMI, 18.5–24.9 kg/m^2^), group 3 (overweight; BMI, 25.0–29.9 kg/m^2^), group 4 (Grade 1 obesity; BMI, 30–34.9 kg/m^2^), group 5 (Grade 2 obesity; BMI, 35–39.9 kg/m^2^), group 6 (Grade 3 obesity; BMI, >40 kg/m^2^).

*determined by 8^*th*^ *edition of AJCC/UICC TNM staging system*

Prognostic factors for extrathyroidal extension were tumor size and multifocality. Prognostic factors for lymph node metastases were age, male gender, tumor diameter, and multifocality. The only prognostic factor for distant metastases was tumor diameter.

BMI was not a statistically significant predictive factor, in contrast to tumor size and multifocality, which were prognostic factors for intermediate and high recurrence risk according to the ATA system. In addition, according to the multivariate logistic regression analysis, BMI was not a predictor of microscopic or gross extrathyroidal extension, either in BMI groups or when BMI was considered as a continuous variable (Table 4). Likewise, BMI was not a statistically significant prognostic factor for poorer clinical response to the primary treatment (indeterminate, biochemically or structurally incomplete) or disease outcome (persistent/recurrent disease or death from cancer) (Table 3).

**Table 4:** Multivariate logistic regression analyses, using BMI groups and BMI as a continuous variable.

	Predictors	Microscopic extrathyroidal extension		Gross extrathyroidal extension	
		OR (95% CI)	p-value	OR (95% CI)	p-value
BMI groups	Age (years)	1.01 (0.99–1.02)	0.200	1.05 (1.02–1.09)	0.003
	Male gender	1.12 (0.68–1.83)	0.652	1.90 (0.74–4.89)	0.184
	Tumor size (mm)	1.03 (1.02–1.04)	<0.001	1.06 (1.04–1.07)	<0.001
	Multifocality	2.17 (1.52–3.08)	<0.001	1.49 (0.60–3.68)	0.391
	BMI	1.04 (0.88–1.23)	0.616	0.95 (0.64–1.43)	0.823
BMI as a continuous variable	Age (years)	1.01 (1.00–1.02)	0.195	1.05 (1.02–1.09)	0.003
	Male gender	1.12 (0.68–1.83)	0.643	1.89 (0.73–4.86)	0.187
	Tumor size (mm)	1.03(1.02–1.04)	<0.001	1.06 (1.04–1.07)	<0.001
	Multifocality	2.17 (1.52–3.08)	<0.001	1.49 (0.60–3.68)	0.388
	BMI	1.01 (0.97–1.04)	0.701	0.95 (0.91–1.07)	0.742

OR: odds ratio; CI: confidence interval; BMI groups: group 1 (underweight; BMI, <18.5 kg/m^2^), group 2 (normal; BMI, 18.5–24.9 kg/m^2^), group 3 (overweight; BMI, 25.0–29.9 kg/m^2^), group 4 (Grade 1 obesity; BMI, 30–34.9 kg/m^2^), group 5 (Grade 2 obesity; BMI, 35–39.9 kg/m^2^), group 6 (Grade 3 obesity; BMI, >40 kg/m^2^).

By contrast, male sex and tumor size were associated with worse response to treatment (indeterminate, biochemically or structurally incomplete), and age and tumor size were prognostic factors for disease outcome (persistent/recurrent disease or death from cancer) (Table 3).

### Overall survival according to BMI group

**Fig 1:**
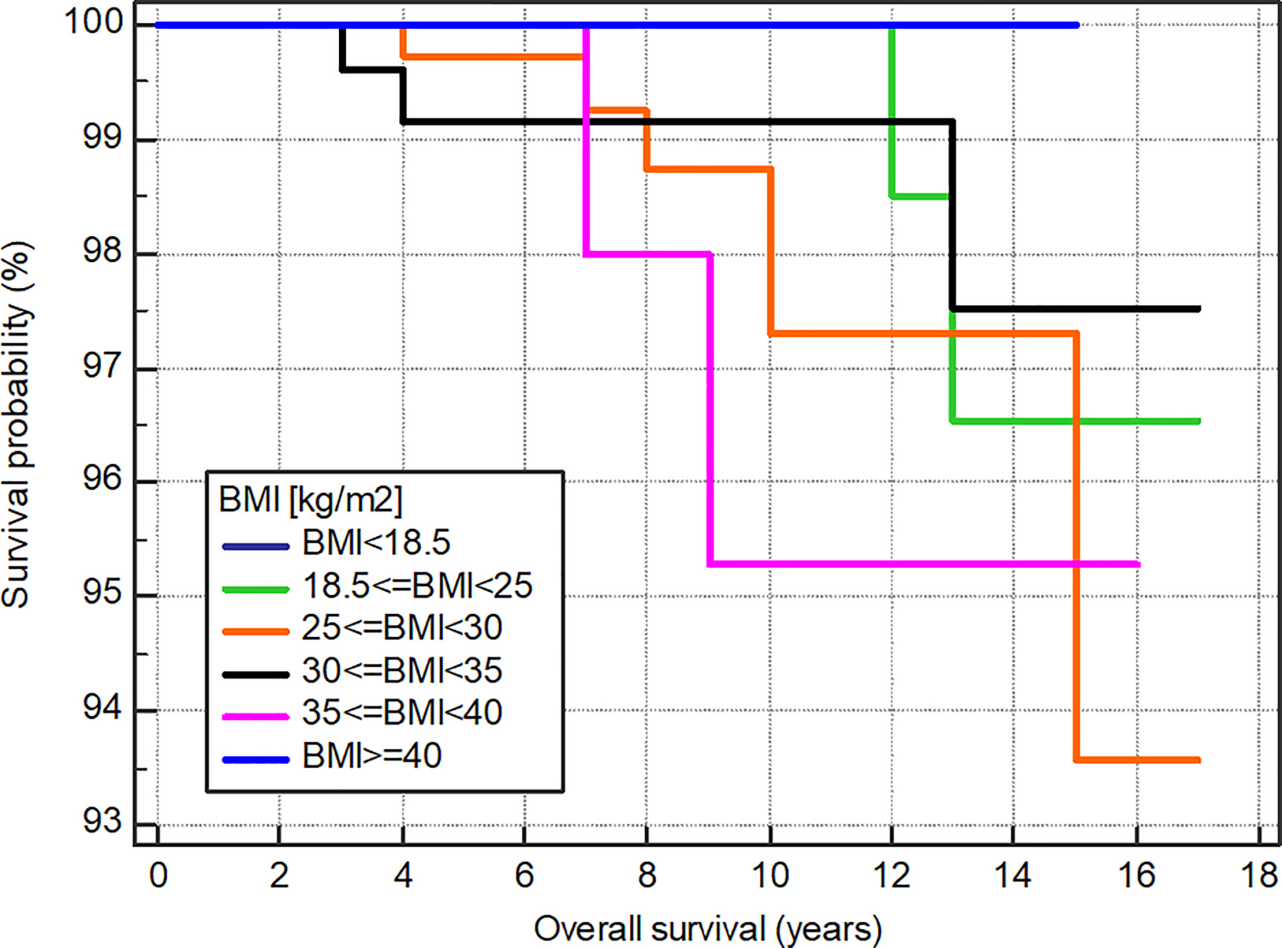
Comparison of overall survival according to BMI group. No significant differences were detected among individuals in the underweight (BMI < 18.5), normal body weight (18.5 ≤ BMI < 25), overweight (25 ≤ BMI < 30), grade 1 obese (30 ≤ BMI < 35), grade 2 obese (35 ≤ BMI < 40), or grade 3 obese (BMI ≥ 40) groups (p = 0.7723).

Median duration of the follow-up of the studied group was 7.7 years (range, 1–16 years). The overall survival was compared between groups using the log-rank test (Fig 1, Table 5). Overall survival did not differ significantly according to BMI (all six groups).

**Table 5:** Comparison of survival curves.

Survival time (years)	BMI < 18.5 n = 8	18.5 ≤ BMI < 25 n = 339	25 ≤ BMI < 30 n = 436	30 ≤ BMI < 35 n = 275	35 ≤ BMI < 40 n = 98	BMI ≥ 40 n = 25
5	100%	100%	99.7%	99.2%	98.0%	100%
10	100%	98.5%	97.3%	99.2%	95.2%	100%
15	100%	96.5%	93.6%	97.5%	95.2%	100%
P-value for log-rank test = 0.7723						

## Discussion

Obesity is a serious global health problem, especially in developed countries, and its prevalence is increasing. It is the cause of many chronic diseases and has been linked to some types of cancer [38–40]. It has been posited that thyroid cancer is related to obesity [18], and the rise in the number of new thyroid cancer cases in recent decades may be due in part to the increased prevalence of obesity [21, 41–43]. However, a causal link between obesity and thyroid cancer is not widely accepted. A retrospective study of fine-needle aspiration biopsies of 4849 thyroid nodules showed no relationship between obesity and cancer risk; the incidence of suspicious or malignant nodules did not differ between five BMI groups (normal body weight, overweight, and Grade 1–3 obesity) [44]. Similarly, no association was found between obesity and thyroid cancer in a study of people undergoing preventive screening for various risk factors for thyroid cancer [45], nor in one other cohort study [24, 46].

In addition to studies on the association of obesity with the incidence of thyroid cancer, several studies have investigated the role of obesity in the aggressiveness of the course of the disease. The results of these studies are mixed, with some studies showing a positive relationship [47–50] and others showing no relationship [26, 27, 51]. In the current study of patients with DTC, there was no relationship between BMI and aggressive clinicopathological features, the degree of clinical progression, the response to primary treatment, or the outcome of the disease. In addition, BMI was not a significant predictor of high or intermediate risk of recurrence according to the ATA Risk Stratification system, as reported by Grani et al. [52]. Our results are in line with those of Kwon et al. [51], in which the authors did not find any association between BMI and clinicopathological features of thyroid cancer or disease outcome. Kim et al. reported that there was no independent association between BMI and stage of PTC at diagnosis [27]. Paes et al. showed no relationship between BMI and aggressive clinicopathological features of thyroid cancer or disease outcome (recurrent/persistent disease) [26]. In the Paes et al. study, the majority of patients were Caucasian (93%), with a median BMI of 27.8 kg/m^2^ [26]. In the present study, the median BMI was similar (28.1 kg/m^2^), and all patients included in the study were Caucasian. By contrast, Kim et al. showed that higher BMI was significantly associated with large tumor size, extrathyroidal extension, and more advanced stage of cancer [48]. In the Kim et al. study, median BMI was 23.8 kg/m^2^ and all patients were Korean. As in the Paes et al. and Kim et al. studies [26, 48], no correlation was found between higher BMI and recurrent/persistent disease, despite differences in clinicopathological features that are known prognostic factors for DTC. Discrepancies between the results of these studies probably arise from the number of obese patients (BMI ≥30 kg/m^2^) enrolled in each study; 101/259 (38.9%) patients in the Paes et al. study were obese, and 398/1181 patients (33.7%) were obese in the present study [26], but only 95/2057 (4.6%) patients in the Kim et al. study were obese [48]. Findings consistent with those of Kim et al. were also obtained in studies performed in China [48, 53, 54]. Paes et al., like the present study, lacked data on such parameters as TSH, fasting glucose, and total cholesterol [26]. The composition of our study population was similar in race and range of obesity to that of the Paes et al. study, which may contribute to the similarity of our research results [26]. Differences pertaining to the small number of obese Asians may result from specific ethnic features of this group. Asians are typically of shorter height and less obese, and their typical diet differs from that of Caucasians [49]. Differences may also result from the type of obesity, duration of obesity, and differences in physical activity. Moreover, the use of the same WHO classification for the Caucasian and Asian populations, as well as the difference in BMI distributions, may be responsible for the conflicting results [54]. These features may explain, among others, discrepancies regarding the relationship between BMI and prognostic factors for thyroid cancer. Consequently, the relationship between BMI and clinicopathological features of thyroid cancer remains controversial.

Another factor that should be taken into account when considering the role of obesity in thyroid cancer is the potential for a delay in diagnosis due to difficulties in detecting thyroid nodules during neck examinations of obese patients. Although the present study and others did not find increased BMI to be associated with larger tumor [26, 51, 52], other studies have observed this trend [47, 48, 55]. Additionally, Tresallet et al. reported that obese patients with PTC >10 mm had an increased risk of persistent / recurrent disease (OR = 3.8, 95% CI: 1.6–8.8; p = 0.03)[55]. However, in this study and in Chung et al. and Kwon et al., no such relationship was observed [51, 56]. In our study, we analyzed the effects of tumor diameter > 10 mm and the tumor diameter considered as a continuous variable on disease outcome in patients with BMI <30 kg/m^2^ and BMI ≥30 kg/m^2^. We observed an increased risk of persistent/recurrent disease in patients with tumor diameter >10 mm, and as a function of tumor diameter when used as a continuous variable in both groups (S2 Table). Thus, the size of the tumor itself, in both obese and non-obese patients, is a strong prognostic factor affecting disease outcome.

In univariate and multivariate analysis, higher BMI was not a predictor of aggressive clinicopathological features of DTC [extrathyroidal extension (microscopic or gross), lymph node metastases, and distant metastases]. Many studies report that BMI is a predictor of microscopic extrathyroidal extension, but our findings and those of Kwon et al. do not confirm this conclusion [48, 52–54, 57]. In our study, tumor size and multifocality were prognostic factors for microscopic extrathyroidal extension. According to the 8^th^ edition of AJCC/TNM staging system, microscopic extrathyroidal extension does not affect cancer stage [58]. Also, higher BMI was not a prognostic factor for a poorer treatment response (indeterminate, biochemically incomplete, or structurally incomplete), nor for a worse outcome of the disease (persistent/recurrent disease or death from cancer). Our results agree with those of Chung et al. [56], in which the authors did not find a significant difference between BMI groups in the outcome of the disease. They found that BMI was not a prognostic factor for PTC, which we also found in the present study.

We investigated the relationship between BMI and the survival of DTC patients and found that there was no significant difference in the overall survival of patients in relation to BMI groups. These results are in line with those of Yousif Al-Ammar et al. [59].

This retrospective study has several strengths. Firstly, it contains a large, ethnically homogeneous ethnic group of patients diagnosed and treated at a single center in Poland, in accordance with current guidelines for thyroid cancer. Secondly, this study included a larger group of obese patients (398 obese out of a total 1181 patients) than previous studies, with more Grade 2 and Grade 3 obese patients (BMI, ≥35 kg/m^2^; n = 123) [26, 48, 52, 55]. Because of this, we were able to perform separate analysis of six different BMI groups for the first time. Lastly, our follow-up period was approximately 7.7 years, similar to that in the work of Kwon et al. (approximately 8.4 years), and longer than those of several other studies [26, 51, 55, 56].

This work also has some limitations. Firstly, because this was a retrospective study, no information was available regarding the duration of obesity and thyroid cancer, waist-to-hip ratio, body fat percentage, skin fold thickness, and abdominal fat evaluation. Thus, this study defined obesity based solely on BMI, similar to many other studies [26, 48, 49, 51, 52, 55, 56]. For most patients, there was no information regarding TSH at the time of the cancer diagnosis available, so the relationship between TSH and thyroid status at the time of the diagnosis could not be assessed. There was also no detailed information on comorbidities such as diabetes, insulin resistance, and hypercholesterolemia, or lifestyle factors such as nutrition, smoking, alcohol consumption, and physical activity.

In conclusion, obesity was not associated with more aggressive clinicopathological features of thyroid cancer in our study. Obesity was not a risk factor for more advanced stages of cancer, nor was it a prognostic factor for poorer treatment response or worse clinical outcome in DTC patients.

## Supporting information

S1 Table (DOCX)

S2 Table (DOCX)

S1 Dataset (DOCX)

S2 Dataset (DOCX)
