# Supplementary material for: The impact of BMI on clinical progress, response to treatment, and disease course in patients with differentiated thyroid cancer
Source: PLoS One. 2018 Oct 1;13(10):e0204668. doi: 10.1371/journal.pone.0204668 (PMC6166948; doi:10.1371/journal.pone.0204668)
Supplement: S1 Table — (DOCX) [file pone.0204668.s001.docx]

**Chi-squared test**

| Classification X | BMI___30 |
| --- | --- |
| Classification Y | Płeć_0_1 |

|  | BMI___30 | |  |
| --- | --- | --- | --- |
| Płeć_0_1 | 0 | 1 |  |
| 0 | 700 67,4% RT 89,4% CT 59,3% GT | 339 32,6% RT 85,2% CT 28,7% GT | 1039 (88,0%) |
| 1 | 83 58,5% RT 10,6% CT 7,0% GT | 59 41,5% RT 14,8% CT 5,0% GT | 142 (12,0%) |
|  | 783 (66,3%) | 398 (33,7%) | 1181 |

RT: % of Row Total; CT: % of Column Total; GT: % of Grand Total

Show all percentages

**Chi-squared test**

| Chi-squared | 4,447 |
| --- | --- |
| DF | 1 |
| Significance level | P = 0,0350 |
| Contingency coefficient | 0,061 |

| [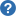](cmd:HELP) [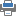](cmd:PRINT) | [Frequency chart](cmd:FREQUENCYCHART) |
| --- | --- |
