# Supplementary material for: The impact of BMI on clinical progress, response to treatment, and disease course in patients with differentiated thyroid cancer
Source: PLoS One. 2018 Oct 1;13(10):e0204668. doi: 10.1371/journal.pone.0204668 (PMC6166948; doi:10.1371/journal.pone.0204668)
Supplement: S2 Table — (DOCX) [file pone.0204668.s002.docx]

Supplementary table. The influence of the size of the dominant tumor on persistent/recurrent disease in groups of obese and not obese patients.

| Variable: | non-obese patients, n=783  BMI <30kg/m^2^ | | obese patients, n=398  BMI ≥30kg/m^2^ | |
| --- | --- | --- | --- | --- |
|  | OR (95% CI) | p-value | OR (95% CI) | p-value |
| Tumor size (mm) | 1.05 (1.04 – 1.07) | <0.001 | 1.04 (1.02 – 1.06) | <0.001 |
| Tumor size >10 mm | 6.92 (3.36 – 14.3) | <0.001 | 4.91 (1.73 – 13.9) | <0.003 |

*OR- odds ratio; BMI- body mass index; CI- confidence interval*
