# Supplementary material for: The impact of BMI on clinical progress, response to treatment, and disease course in patients with differentiated thyroid cancer
Source: PLoS One. 2018 Oct 1;13(10):e0204668. doi: 10.1371/journal.pone.0204668 (PMC6166948; doi:10.1371/journal.pone.0204668)
Supplement: S1 Dataset — (DOCX) [file pone.0204668.s003.docx]

**Chi-squared test**

| Classification X | BMI_6_grup |
| --- | --- |
| Classification Y | TNM_I_II_vs._III_IV_8_edycja |
| Filter | Wiek_55=1 |

|  | BMI_6_grup | | | | |  |
| --- | --- | --- | --- | --- | --- | --- |
| TNM_I_II_vs._III_IV_8_edycja | 2 | 3 | 4 | 5 | 6 |  |
| 0 | 81 17,4% RT 92,0% CT 16,2% GT | 197 42,4% RT 93,4% CT 39,5% GT | 125 26,9% RT 92,6% CT 25,1% GT | 51 11,0% RT 94,4% CT 10,2% GT | 11 2,4% RT 100,0% CT 2,2% GT | 465 (93,2%) |
| 1 | 7 20,6% RT 8,0% CT 1,4% GT | 14 41,2% RT 6,6% CT 2,8% GT | 10 29,4% RT 7,4% CT 2,0% GT | 3 8,8% RT 5,6% CT 0,6% GT | 0 0,0% RT 0,0% CT 0,0% GT | 34 (6,8%) |
|  | 88 (17,6%) | 211 (42,3%) | 135 (27,1%) | 54 (10,8%) | 11 (2,2%) | 499 |

RT: % of Row Total; CT: % of Column Total; GT: % of Grand Total

Show all percentages

**Chi-squared test**

| Chi-squared | 1,205 |
| --- | --- |
| DF | 4 |
| Significance level | P = 0,8773 |

**Chi-squared test for trend**

| Chi-squared (trend) | 0,489 |
| --- | --- |
| DF | 1 |
| Significance level | P = 0,4842 |

|  |  |
| --- | --- |

**Chi-squared test**

| Classification X | BMI_6_grup |
| --- | --- |
| Classification Y | TNM_8_edycja |
| Filter | Wiek_55=0 AND (TNM_8_edycja<=2) |

|  | BMI_6_grup | | | | | |  |
| --- | --- | --- | --- | --- | --- | --- | --- |
| TNM_8_edycja | 1 | 2 | 3 | 4 | 5 | 6 |  |
| 1 | 8 1,2% RT 100,0% CT 1,2% GT | 247 36,9% RT 98,4% CT 36,2% GT | 222 33,2% RT 98,7% CT 32,6% GT | 137 20,5% RT 97,9% CT 20,1% GT | 42 6,3% RT 93,3% CT 6,2% GT | 13 1,9% RT 100,0% CT 1,9% GT | 669 (98,1%) |
| 2 | 0 0,0% RT 0,0% CT 0,0% GT | 4 30,8% RT 1,6% CT 0,6% GT | 3 23,1% RT 1,3% CT 0,4% GT | 3 23,1% RT 2,1% CT 0,4% GT | 3 23,1% RT 6,7% CT 0,4% GT | 0 0,0% RT 0,0% CT 0,0% GT | 13 (1,9%) |
|  | 8 (1,2%) | 251 (36,8%) | 225 (33,0%) | 140 (20,5%) | 45 (6,6%) | 13 (1,9%) | 682 |

RT: % of Row Total; CT: % of Column Total; GT: % of Grand Total

Show all percentages

**Chi-squared test**

| Chi-squared | 6,430 |
| --- | --- |
| DF | 5 |
| Significance level | P = 0,2666 |

**Chi-squared test for trend**

| Chi-squared (trend) | 1,829 |
| --- | --- |
| DF | 1 |
| Significance level | P = 0,1763 |

|  |  |
| --- | --- |
