# Supplementary material for: The impact of BMI on clinical progress, response to treatment, and disease course in patients with differentiated thyroid cancer
Source: PLoS One. 2018 Oct 1;13(10):e0204668. doi: 10.1371/journal.pone.0204668 (PMC6166948; doi:10.1371/journal.pone.0204668)
Supplement: S2 Dataset — (DOCX) [file pone.0204668.s004.docx]

1. There was no significant correlation between BMI [kg/m2] and:

a) dominant tumor diameter (spearman r = 0.019, p = 0.5157)
b) ATA as low, intermediate, high (r = 0.02, p = 0.4898).

2. There was no significant relationship between BMI [kg/m2] from:

a) Lymph node metastasis (Z = 1.14, p = 0.2543),
b) Distant metastasis (t = 0.05, p = 0.9611),
c) primary response as excellent, intermediate, biochemically incomplete, structurally
 incomplete (t = 1.01, p = 0.8008)
d) status of final follow-up as remission vs. recurrent/persistent disease (t = 4.32, p = 0.151),
e) vascular invasion as yes vs. no (Z=0.039; p=0.9693)
f) extrathyroidal extension as negative, microscopic, macroscopic (t=2.75; p=0.2527)

**1 a) Correlation table -** dominant tumor diameter (spearman r = 0.019, p = 0.5157)

| Variable Y | dominant tumor diameter |
| --- | --- |
| Variable X | BMI |

| Sample size | 1181 |
| --- | --- |
| Correlation coefficient r | 0,02299 |
| Significance level | P=0,4339 |
| 95% Confidence interval for r | -0,03459 to 0,08042 |

|  |  |
| --- | --- |

# 1 b) Rank Correlation - ATA as low, intermediate, high (r = 0.02, p = 0.4898).

| Variable Y | ATA 0 (low), 1 (intermediate) 2 (high) |
| --- | --- |
| Variable X | BMI |

| Sample size | 1181 |
| --- | --- |
| Spearman's coefficient of rank correlation (rho) | 0,0201 |
| Significance level | P=0,4898 |
| 95% Confidence Interval for rho | -0,0370 to 0,0771 |

|  |  |
| --- | --- |

**2 a) Lymph node metastasis (Z = 1.14, p = 0.2543),**

**Summary statistics table**

|  | BMI | |
| --- | --- | --- |
| N01- **Lymph node metastasis** | 0 | 1 |
| N | 1044 | 137 |
| Minimum | 16,650 | 17,850 |
| Maximum | 53,150 | 39,780 |
| Mean | 28,194 | 27,559 |
| Median | 27,780 | 27,240 |
| SD | 5,1362 | 5,1900 |
| 25 - 75 P | 24,390 to 31,395 | 23,583 to 30,865 |
| Normal Distr. | <0,0001 | 0,0455 |

# Mann-Whitney test (independent samples)

| Sample 1 | |
| --- | --- |
| Variable | BMI |
| Filter | N01=0 |
| Sample 2 | |
| Variable | BMI |
| Filter | N01=1 |

|  | Sample 1 | Sample 2 |
| --- | --- | --- |
| Sample size | 1044 | 137 |
| Lowest value | [16,6500](cmd:SHOWXMINMAX?1) | [17,8500](cmd:SHOWYMINMAX?4) |
| Highest value | [53,1500](cmd:SHOWXMINMAX?1181) | [39,7800](cmd:SHOWYMINMAX?1153) |
| Median | 27,7800 | 27,2400 |
| 95% CI for the median | 27,4384 to 28,1031 | 26,3275 to 28,4907 |
| Interquartile range | 24,3900 to 31,3950 | 23,5825 to 30,8650 |

| Hodges-Lehmann median difference | -0,5500 |
| --- | --- |
| 95% Confidence interval | -1,5100 to 0,3900 |

## Mann-Whitney test (independent samples)

| Average rank of first group | 595,0982 |
| --- | --- |
| Average rank of second group | 559,7701 |
| Mann-Whitney U | 67235,50 |
| **Test statistic Z (corrected for ties)** | **1,140** |
| **Two-tailed probability** | **P = 0,2543** |

|  |  |
| --- | --- |

**b) Distant metastasis (Z =0,0487, p = 0.9611),**

# Summary statistics table

|  | BMI | |
| --- | --- | --- |
| M01 | 0 | 1 |
| N | 1160 | 21 |
| Minimum | 16,650 | 19,330 |
| Maximum | 53,150 | 37,550 |
| Mean | 28,126 | 27,800 |
| Median | 27,690 | 29,240 |
| SD | 5,1494 | 4,9578 |
| 25 - 75 P | 24,250 to 31,345 | 23,965 to 30,855 |
| Normal Distr. | <0,0001 | 0,4224 |

# Mann-Whitney test (independent samples)

| Sample 1 | |
| --- | --- |
| Variable | BMI |
| Filter | M01=0 Distant metastasis - no |
| Sample 2 | |
| Variable | BMI |
| Filter | M01=1 Distant metastasis - yes |

|  | Sample 1 | Sample 2 |
| --- | --- | --- |
| Sample size | 1160 | 21 |
| Lowest value | [16,6500](cmd:SHOWXMINMAX?1) | [19,3300](cmd:SHOWYMINMAX?22) |
| Highest value | [53,1500](cmd:SHOWXMINMAX?1181) | [37,5500](cmd:SHOWYMINMAX?1129) |
| Median | 27,6900 | 29,2400 |
| 95% CI for the median | 27,3839 to 28,0400 | 25,0513 to 30,6824 |
| Interquartile range | 24,2500 to 31,3450 | 23,9650 to 30,8550 |

| Hodges-Lehmann median difference | -0,04000 |
| --- | --- |
| 95% Confidence interval | -2,3600 to 2,2500 |

## Mann-Whitney test (independent samples)

| Average rank of first group | 591,0651 |
| --- | --- |
| Average rank of second group | 587,4048 |
| Mann-Whitney U | 12104,50 |
| **Test statistic Z (corrected for ties)** | **0,0487** |
| **Two-tailed probability** | **P = 0,9611** |

|  |  |
| --- | --- |

**c) primary response as excellent, intermediate, biochemically incomplete, structurally
 incomplete (t = 1.01, p = 0.8008)**

# Summary statistics table

|  | BMI | | | |
| --- | --- | --- | --- | --- |
| primary response | excellent | intermediate | biochemically incomplete | structurally   incomplete |
| N | 991 | 108 | 27 | 55 |
| Minimum | 16,650 | 19,050 | 17,850 | 19,330 |
| Maximum | 53,150 | 46,410 | 36,810 | 39,780 |
| Mean | 28,092 | 28,463 | 28,473 | 27,792 |
| Median | 27,680 | 28,140 | 28,670 | 27,780 |
| SD | 5,1414 | 5,3758 | 5,4694 | 4,6438 |
| 25 - 75 P | 24,182 to 31,307 | 24,645 to 31,610 | 24,872 to 32,890 | 24,425 to 30,540 |
| Normal Distr. | <0,0001 | 0,0465 | 0,4257 | 0,3400 |

# Kruskal-Wallis test

| Data | BMI |
| --- | --- |
| Factor codes | primary response |

| Sample size | 1181 |
| --- | --- |

## Descriptive statistics

| Factor | n | Minimum | 25th percentile | Median | 75th percentile | Maximum |
| --- | --- | --- | --- | --- | --- | --- |
| exelent | 991 | 16,6500 | 24,182 | 27,680 | 31,307 | 53,150 |
| intermediate | 108 | 19,0500 | 24,645 | 28,140 | 31,610 | 46,410 |
| Biochemically incomplete | 27 | 17,8500 | 24,872 | 28,670 | 32,890 | 36,810 |
| Structurally incomplete | 55 | 19,3300 | 24,425 | 27,780 | 30,540 | 39,780 |

## Kruskal-Wallis test

| **Test statistic** | **1,0017** |
| --- | --- |
| Corrected for ties  Ht | 1,0017 |
| Degrees of Freedom (DF) | 3 |
| **Significance level** | **P = 0,800846** |

| Factor | n | Average Rank |
| --- | --- | --- |
| (1) exelent | 991 | 588,64 |
| (2) intermediate | 108 | 610,00 |
| (3) Biochemically incomplete | 27 | 636,54 |
| (4) Structurally incomplete | 55 | 573,93 |

|  |  |
| --- | --- |

**d) status of final follow-up as remission vs. recurrent/persistent disease (Z=1.26, p= 0.2061),**

# Summary statistics table

|  | BMI | |
| --- | --- | --- |
| status of final follow-up | remission | recurrent/persistent |
| N | 1097 | 84 |
| Minimum | 16,650 | 17,850 |
| Maximum | 53,150 | 41,560 |
| Mean | 28,074 | 28,727 |
| Median | 27,680 | 27,925 |
| SD | 5,1438 | 5,1420 |
| 25 - 75 P | 24,170 to 31,257 | 25,560 to 31,745 |
| Normal Distr. | <0,0001 | 0,5248 |

# Mann-Whitney test (independent samples)

| Sample 1 | |
| --- | --- |
| Variable | BMI |
| Filter | status of final follow-up = remission |
| Sample 2 | |
| Variable | BMI |
| Filter | status of final follow-up = recurrent/persistent |

|  | Sample 1 | Sample 2 |
| --- | --- | --- |
| Sample size | 1097 | 84 |
| Lowest value | [16,6500](cmd:SHOWXMINMAX?1) | [17,8500](cmd:SHOWYMINMAX?4) |
| Highest value | [53,1500](cmd:SHOWXMINMAX?1181) | [41,5600](cmd:SHOWYMINMAX?1172) |
| Median | 27,6800 | 27,9250 |
| 95% CI for the median | 27,2800 to 28,0580 | 27,1963 to 29,7246 |
| Interquartile range | 24,1700 to 31,2575 | 25,5600 to 31,7450 |

| Hodges-Lehmann median difference | 0,7500 |
| --- | --- |
| 95% Confidence interval | -0,4100 to 1,9200 |

## Mann-Whitney test (independent samples)

| Average rank of first group | 587,5273 |
| --- | --- |
| Average rank of second group | 636,3512 |
| Mann-Whitney U | 42264,50 |
| **Test statistic Z (corrected for ties)** | **1,264** |
| **Two-tailed probability** | **P = 0,2061** |

|  |  |
| --- | --- |

**e) vascular invasion as yes vs. no (Z=0.039; p=0.9693)**

# Summary statistics table

|  | BMI | |
| --- | --- | --- |
| vascular invasion | no | yes |
| N | 1111 | 70 |
| Minimum | 16,650 | 19,330 |
| Maximum | 53,150 | 39,780 |
| Mean | 28,124 | 28,060 |
| Median | 27,730 | 27,730 |
| SD | 5,1727 | 4,7030 |
| 25 - 75 P | 24,220 to 31,387 | 25,040 to 30,120 |
| Normal Distr. | <0,0001 | 0,2655 |

# Mann-Whitney test (independent samples)

| Sample 1 | |
| --- | --- |
| Variable | BMI |
| Filter | vascular invasion ="no" |
| Sample 2 | |
| Variable | BMI |
| Filter | vascular invasion ="yes" |

|  | Sample 1 | Sample 2 |
| --- | --- | --- |
| Sample size | 1111 | 70 |
| Lowest value | [16,6500](cmd:SHOWXMINMAX?1) | [19,3300](cmd:SHOWYMINMAX?23) |
| Highest value | [53,1500](cmd:SHOWXMINMAX?1181) | [39,7800](cmd:SHOWYMINMAX?1153) |
| Median | 27,7300 | 27,7300 |
| 95% CI for the median | 27,3537 to 28,0900 | 26,2200 to 29,6111 |
| Interquartile range | 24,2200 to 31,3875 | 25,0400 to 30,1200 |

| Hodges-Lehmann median difference | 0,02000 |
| --- | --- |
| 95% Confidence interval | -1,1600 to 1,2500 |

## Mann-Whitney test (independent samples)

| Average rank of first group | 590,9041 |
| --- | --- |
| Average rank of second group | 592,5214 |
| Mann-Whitney U | 38778,50 |
| **Test statistic Z (corrected for ties)** | **0,0385** |
| **Two-tailed probability** | **P = 0,9693** |

|  |  |
| --- | --- |

**f) extrathyroidal extension as negative, microscopic, macroscopic (t=2.75; p=0.2527)**

# Summary statistics table

|  | BMI | | |
| --- | --- | --- | --- |
| extrathyroidal extension | negative | microscopic | macroscopic |
| N | 955 | 191 | 35 |
| Minimum | 17,570 | 16,650 | 20,310 |
| Maximum | 53,150 | 46,410 | 37,550 |
| Mean | 28,019 | 28,551 | 28,545 |
| Median | 27,610 | 28,000 | 28,040 |
| SD | 5,1982 | 4,9461 | 4,6919 |
| 25 - 75 P | 24,133 to 31,247 | 25,160 to 31,777 | 24,967 to 31,505 |
| Normal Distr. | <0,0001 | 0,0172 | 0,5087 |

# Kruskal-Wallis test

| Data | BMI |
| --- | --- |
| Factor codes | extrathyroidal extension |

| Sample size | 1181 |
| --- | --- |

## Descriptive statistics

| Factor | n | Minimum | 25th percentile | Median | 75th percentile | Maximum |
| --- | --- | --- | --- | --- | --- | --- |
| 0 | 955 | 17,5700 | 24,133 | 27,610 | 31,247 | 53,150 |
| 1 | 191 | 16,6500 | 25,160 | 28,000 | 31,777 | 46,410 |
| 2 | 35 | 20,3100 | 24,967 | 28,040 | 31,505 | 37,550 |

## Kruskal-Wallis test

| **Test statistic** | **2,7514** |
| --- | --- |
| Corrected for ties  Ht | 2,7514 |
| Degrees of Freedom (DF) | 2 |
| **Significance level** | **P = 0,252667** |

| Factor | n | Average Rank |
| --- | --- | --- |
| (1) 0 | 955 | 583,03 |
| (2) 1 | 191 | 623,13 |
| (3) 2 | 35 | 633,14 |

|  |  |
| --- | --- |
